# Two Distinct Dynamic Modes Subtend the Detection of Unexpected Sounds

**DOI:** 10.1371/journal.pone.0085791

**Published:** 2014-01-27

**Authors:** Jean-Rémi King, Alexandre Gramfort, Aaron Schurger, Lionel Naccache, Stanislas Dehaene

**Affiliations:** 1 Cognitive Neuroimaging Unit, Institut National de la Santé et de la Recherche Médicale (INSERM) U992, Gif/Yvette, France; 2 NeuroSpin Center, Institute of BioImaging Commissariat à l'Energie Atomique (CEA), Gif/Yvette, France; 3 Institut du Cerveau et de la Moelle Épinière Research Center (ICM), Institut National de la Santé et de la Recherche Médicale (INSERM), U975, Paris, France; 4 Institut Mines-Télécom, Télécom ParisTech, Centre National de la Recherche Scientifique (CNRS) Laboratoire Traitement et Communication de l'Information (LTCI), Paris, France; 5 Parietal, Institut National de Recherche en Informatique et en Automatique (INRIA), Commissariat à l'Energie Atomique (CEA), Gif-sur-Yvette Cedex, France; 6 Assistant Publique Hôpitaux de Paris (AP-HP), Groupe hospitalier Pitié-Salpêtrière, Department of Neurophysiology, Paris, France; 7 Faculté de Médecine Pitié-Salpêtrière, Université Paris 6, Paris, France; 8 Université Paris 11, Orsay, France; 9 Collège de France, Paris, France; Max Planck Institute for Human Cognitive and Brain Sciences, Germany

## Abstract

The brain response to auditory novelty comprises two main EEG components: an early mismatch negativity and a late P300. Whereas the former has been proposed to reflect a prediction error, the latter is often associated with working memory updating. Interestingly, these two proposals predict fundamentally different dynamics: prediction errors are thought to propagate serially through several distinct brain areas, while working memory supposes that activity is sustained over time within a stable set of brain areas. Here we test this temporal dissociation by showing how the generalization of brain activity patterns across time can characterize the dynamics of the underlying neural processes. This method is applied to magnetoencephalography (MEG) recordings acquired from healthy participants who were presented with two types of auditory novelty. Following our predictions, the results show that the mismatch evoked by a local novelty leads to the sequential recruitment of distinct and short-lived patterns of brain activity. In sharp contrast, the global novelty evoked by an unexpected sequence of five sounds elicits a sustained state of brain activity that lasts for several hundreds of milliseconds. The present results highlight how MEG combined with multivariate pattern analyses can characterize the dynamics of human cortical processes.

## Introduction

When faced with an unexpected sensory event, the brain must perform two major computations: *i)* identify the most probable reason for the novelty and *ii)* determine whether this novel information is relevant to future decisions. Indeed, when comparing the brain response elicited by expected sounds (“standard”) and unexpected sounds (“deviant”), two radically different electroenphalography (EEG) components are observed: the mismatch negativity (MMN), peaking over centro-anterior EEG sites between ∼100 and 150 ms [1], and the P300 over centro-posterior electrodes [2]. The MMN is primarily generated within superior temporal areas [3]–[6], whereas the P300 involves distributed areas of the frontal, parietal and temporal lobes [7], [8]. The MMN and P300 are also functionally dissociable. The MMN is robust to instructions, subjects' attention, and the subjects' state of consciousness [4], [5], [7], [9]–[15]. Conversely, the full-scale P300 is highly sensitive to whether or not subjects consciously detect the novelty [7], [8], [11]. Finally, whereas any low-level novelty in pitch, duration, or identity triggers an MMN [1], [4], the P300 requires the violation of relevant rules constructed over several seconds [7], [11], [15], [16].

The two EEG components may thus reflect two different computations: the P300 is thought to index a working memory update, passing relevant information to the next trial [8], [17], [18], whereas the MMN would reflect a prediction error signal [4], [19], [20], elicited whenever an incoming stimulus differs from its internally generated prediction [21]–[23].

In previous studies, we have used fMRI [7], EEG [7], [9]–[11], [15], MEG [11], [15] and intracranial recordings [7], [11] to identify the location (*e.g.* “Which brain areas generate the MMN?”) and the timing (*e.g.* “When is the MMN peaking?”) of these different brain responses. A slightly different question relates to their dynamical structure. Crucially, working memory and predictive coding imply fundamentally different dynamics: predictive coding stipulates that errors propagate through a series of different areas until the appropriate internal model cancels the prediction error [21]–[23], while working memory implies an active maintenance of information in a stable activity pattern. In other words, the MMN is predicted to reflect a fast serial process whereas the P300 should reflect a slow and stable activation.

**Figure 1:**
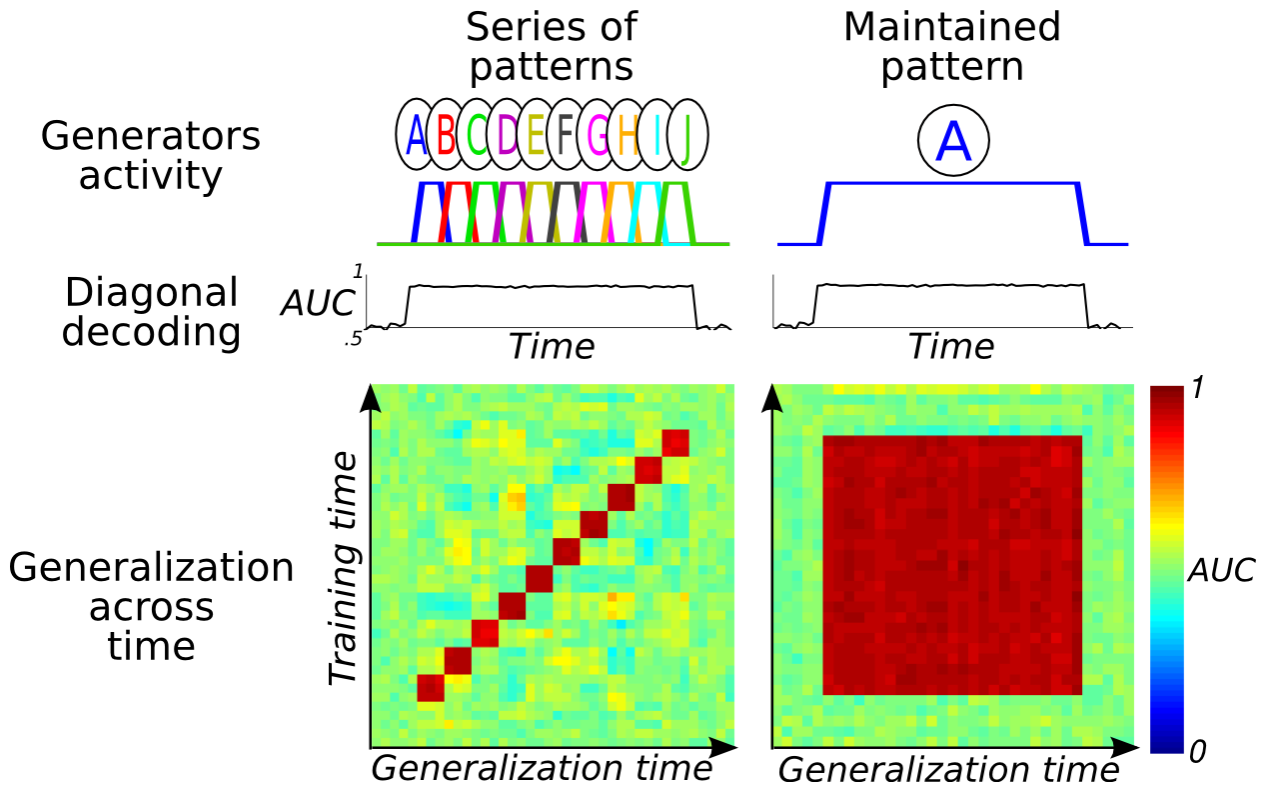
Detecting two types of brain dynamics by assessing the ability of multivariate pattern classifiers to generalize across time. The temporal generalization method can characterize the dynamics of neural activity. (left) When the stimulus evokes a serial chain of brain activations, “diagonal classifiers”, trained and tested at each time point can extract stimulus information throughout the activation period. However, as each classifier is specific to the time point at which it has been trained, they cannot generalize across other time samples. The generalization time analysis thus reveals a diagonal generalization matrix. (right) By contrast, if the underlying activity is sustained over time, then all classifiers would capture the same pattern. These classifiers would thus generalize to one another and lead to a square generalization matrix.

**Figure 2:**
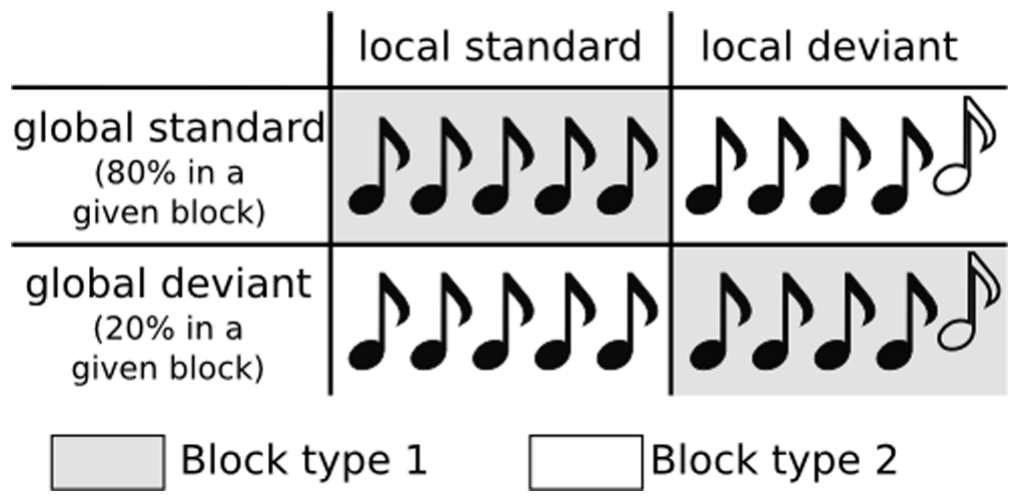
Violating two types of auditory regularities. The Local-Global experimental design [7] is a variation of the auditory oddball task. It consists in presenting series of 5-sound sequences which are composed of five identical sounds (local standard) or four identical sounds followed by a deviant one (local deviant). The global regularity is established across trials by making 80% of the trials identical (global standard). The design thus dissociates the violation of local predictions (change of sound in a given trial) and global predictions (change of sequence across trials).

Here, we put these predictions to a test using magnetoencephalography (MEG) recordings and multivariate decoding. To characterize the two predicted dynamic patterns, a multivariate pattern classifier was first trained to discriminate standard from deviant trials at each time sample. Subsequently, their ability to generalize to new time samples was examined. A *temporal generalization matrix* that can distinguish two types of dynamics is thus obtained (Figure 1). This approach was applied to two different violations of auditory regularities originally designed to isolate the MMN and the P300 components (Figure 2) [7].

## Methods

### Procedure, Material & Apparatus

The Local-Global experimental design [7] enables the comparison of effects engendered by physically identical but contextually different auditory stimuli (Figure 2). Subjects were repeatedly presented to five-sound sequences which were either composed of five identical sounds (xxxxx, “local standard”, LS), or four identical sounds followed by a deviant one (xxxxY, “local deviant”, LD). Trials were presented in two types of blocks, both composed of frequent (“global standard”, GS) and rare trials (“global deviant”, GD) pseudo randomly distributed at least one and at most six global-standard trials apart. In block type 1, 80% of the trials were local standard (LSGS), and 20% were local deviant (LDGD). In block type 2, 80% of the trials were local deviant (LDGS), and the remaining trials were local standard (LSGD). This 2×2 design thus allows dissociating the effect elicited by a local violation (local standard – local deviant) or by a global violation (global standard – global deviant) (Figure 2). Each block was preceded by a ∼30 s habituation phase during which only global standard trials were presented. Habituation trials and trials following a global deviant trial were excluded from the analyses, which were thus based on a total of 780 trials. Other, “omission” trials, that are the focus of a previous study [15], were excluded from the present analyses. Further methodological details can be found in [11], [15].

Each recording session comprised 14 blocks (780 trials) of ∼3.5 minutes duration. Nine healthy volunteers (Age M = 25 years old, SD = 4.7 years, 5 females) were asked to pay attention to the auditory stimuli while keeping their eyes opened and fixated at a central cross. Note that unlike Bekinschtein *et al.* [7]'s original design, subjects were not asked to count the global deviant trials. All subjects gave written informed consent to participate to this study, which was approved by the local Ethics Committee (Comité de protection des personnes “Ile-de-France VII”, hôpital de Bicêtre, 78 rue du Général-Leclerc, 94270 Le Kremlin-Bicêtre). Traditional analyses (topography, sources, *etc.*) have been partially reported in [11], [15].

Signal space separation (SSS, [24]) was applied to suppress external magnetic interference, interpolate noisy MEG sensors and realign MEG data into a subject-specific head position with Maxfilter software application (Elekta Neuromag®). This reference head position was determined from head position measurements acquired at the beginning of each recording session. Eye blink and cardiac artifacts were corrected separately for each type of channel (gradiometer and magnetometers) using signal space projection (SSP, [25]). All signals were digitally low-pass filtered at 40 Hz and down-sampled to 256 Hz. Trials were then segmented from −800 ms to 700 ms after the critical stimulus onset, and were corrected for baseline over a 200 ms window before the onset of the first of the five sounds. Trials with large artifacts remaining after correction for ocular and cardiac artifacts were identified manually and excluded from the present analyses.

### Contrasts and classes

Two types of classifications were attempted (Figure 2): (1) local standard (*n = 390*) versus local deviant trials (*n = 390*); (2) global standard (*n = 600*) versus global deviant trials (*n = 180*). Both of these analyses contrast trials that are evenly distributed across blocks and are therefore free of potential block-design artifacts [26].

### Multivariate pattern analysis (MVPA)

Multivariate pattern analyses (MVPA) were implemented to systematically track the dynamics of neural processes recorded with MEG. Our method is based on the common principle that when a brain area – or set of areas – is activated, its magnetic fields project to the MEG sensors in a specific spatial pattern, and can thus be isolated by a particular topography. The aim of the present MVPA is thus to construct, at each time point and for each subject separately, a classifier that specifically isolate such topography.

The detailed procedure of the multivariate pattern analysis is reported in [11] and a Python script has been made publicly available [27] in the MNE software [28] to test temporal generalization method on a public dataset [29]. A ten-fold stratified cross-validation was implemented for each within-subject analysis. Stratified cross-validation balances the proportion of each class (LSGS, LDGS, LSGD, LDGD) in each fold. For each fold and at each time sample, a linear support vector machine (SVM, [30]) was fit on 9/10 of the trials (training set) with a single time sample recorded across the 306 MEG sensors. MEG signals were normalized (subtract mean and divide by standard deviation) within the cross-validation and for each classifier separately. No dimensionality reduction or feature selection was applied here, as the number of features remained relatively low (n = 306). Each SVM aimed at finding the hyperplane (*w*, *i.e.* the topography) that best discriminated standard and deviant trials at each time sample. Note that because the SVM kernel is linear and because the SVM features are the amplitude recorded in MEG channels, the present analysis can only capture brain waves that are phase-locked to the auditory stimulation (*i.e.* evoked but not induced brain activations [31]). Following previous analyses [11], the regularization parameter (C) was fixed to 1. The SVM was supplemented with Platt's method [32] that provides a continuous, probabilistic, estimate (e.g. continuous prediction: probability of being deviant) rather than a categorical output (discrete prediction: either deviant or local standard). Classification performance was then computed with a Received Operative Curve (ROC), based on the probabilistic classification of an independent test set (1/10). Finally, a sample weighting procedure was applied in proportion to the classes (LSGS, LSGD, LDGS, LDGD) so as to equalize the contribution of each of these categories in the definition of the *w*. All multivariate analyses were performed with the Scikit-Learn toolbox [33].

As discussed elsewhere (*e.g.* [34]–[36]), cross-validation methods can be less sensitive than classical inferences, partly because each fitting procedure is trained on a subset of the data. However, the multidimensional distribution of the present data being unknown and the number of samples (*i.e.* number of trials for each subject) being relatively small compared to the number of features (*i.e.* number of channels), the assumptions of traditional multivariate inference statistics (*e.g.* MANOVA) would not hold. Moreover, cross-validating is here particularly important because MEG signals are auto-correlated. As a consequence, training a classifier on a set of trials at time *t* could potentially generalize to *t′* only because of auto-correlated noise, and therefore even in the absence of information.

#### Generalization across Time

Crucially, each classifier is not only assessed on its ability to decode information at the time point at which it has been trained, but is also assessed on its ability to generalize across other time samples. The principle of the present temporal generalization method is similar to the one employed in previous multi-unit recording studies, in which one or several patterns of neuronal activity are first isolated with a linear classifier at a particular time window and then tracked over time (*e.g.* [37]–[41]). Recently, similar approaches have also been used with MEG recordings (*e.g.* [42]–[45]). Once *t* linear classifiers have been fitted (where *t* is the duration of a trial expressed in time samples), each of these classifiers is tested on its ability to discriminate the two types of trials at any time *t′*. This method thus leads to a *temporal generalization matrix* of training time *x* testing time. In each cell of the matrix, decoding performance is summarized by the Area Under the Curve (AUC). Classifiers trained and tested at the same time point correspond to the diagonal of this *t^2^* matrix, and are thus referred to as “diagonal” decoding. The decoding performance obtained when *t′* differ from *t* is referred to as “off-diagonal” decoding. Note that the cross-validation was applied independently of the temporal generalization analyses: the trials used in the training set at time t were never included in the generalization at time t′ as consecutive time samples are not independent. Simple simulations are detailed below in order to clarify the aim of this analysis.

To compute the average duration over which temporal generalization remained significant, we computed the number of time samples during which each classifier could significantly predict the trials' classes, using false discovery rate (FDR) to correct for multiple comparison. To avoid underestimating the mean generalization time, we only considered the time window during which the diagonal classifiers performed above chance (82 ms–450 ms).

#### Statistics & Effect sizes

To test for statistical significance within subjects, Mann-Whitney U tests were performed on the classifiers' continuous outputs, with trials as the random variable. Similarly, across-subjects statistics were performed using Wilcoxon Signed Rank Tests. Effect sizes are summarized with the AUC computed from empirical ROC analyses. An AUC of 50% implies that true positive predictions (*e.g.* trial was correctly predicted to belong to class α) and false positive predictions (*e.g.* trial was erroneously predicted to belong to class α) are, on average, equally probable; an AUC of 100% indicates a perfect prediction with no false positives. In principle, for the diagonal decoding, classification performance should not yield AUCs that are significantly below 50%. However, when a classifier fitting and testing time differ, AUCs can be significantly below 50%, as the pattern of brain activation carrying the discriminative information can be flipped in sign between *t* and *t′*. Statistical analyses were performed with MATLAB 2009b.

Two common yet important statistical points may be worth noting here. First, statistical significance (*i.e.* p-value) is related but distinct from classification performance (*i.e.* AUC). Indeed, while the former indicates whether the test is likely to reflect a non-random result (*i.e.* “Is there decodable information?”), the latter indicates the extent to which each trial can be classified from MEG signals (*i.e.* “How much information is there?”). Second, the use of non-parametric statistical methods was motivated by the non-Gaussian distribution of our data (see [11]'s supplementary materials).

#### Simulations

A series of simulations were generated to test the principle of the temporal generalization method. For each class, 50 trials (50% in each class) were generated across 20 simulated sensors and 80 time-samples (*t*). Each generator (*g*), simulating one or several brain areas, projected on a random combination of sensors (*C*), and was activated (*A*) with a temporal profile specific to each simulation. Each generator was thus defined by a vector of 20×1 features of normally distributed values, as well as by a second vector of *t* time-samples indicative of its activity. Each trial (*S(c,t)*) corresponded to the sum of the generators' activities in the direction of the class (class *y* = [−1, 1]):
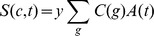



Subsequently, Gaussian white noise was added to all signals. Signal-to-noise ratio was set to 0.5. Finally, each simulation was repeated ten times to simulate a group of subjects. In the simulation of a sequential pattern, 10 generators were successively active for six time samples each. In the simulation of sustained brain activity, a single generator was active for 60 time samples (Figure 1).

## Results

The generalization-across-time analyses were applied to nine subjects who performed the Local-Global task while their brain activity was recorded with MEG.

**Figure 3:**
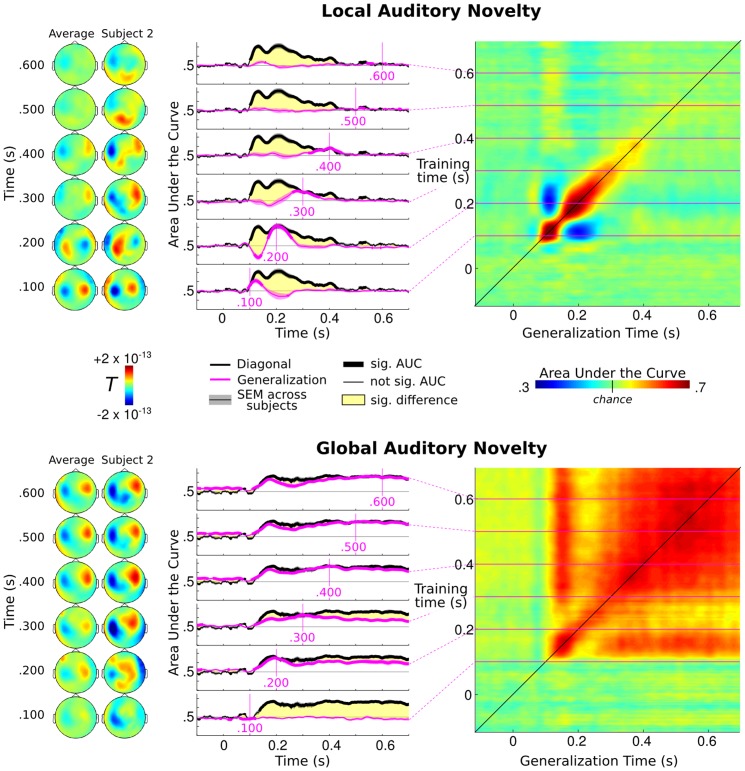
Generalization across time of the local and global responses to auditory novelty. At each time point, a classifier was trained to extract the pattern of MEG activity that distinguishes local-standard from local-deviant trials (mismatch effect, top) or to contrast global-standard from global-deviant trials (bottom). Each classifier was subsequently tested on its ability to generalize this discrimination to all other time samples. (left) Differential patterns (standard – deviant) of brain activity across subjects as well as in a single representative subject using classic univariate analyses. For simplicity purposes, only the magnetometers are plotted (n = 102/306 channels). Note that, unlike subject-specific decoding, classic event related fields (ERF) analyses are tested across subjects, and are thus insensitive to inter-individual variability of subjects' topographies. (middle) Generalization of six different classifiers trained at regularly spaced times between 100 ms and 600 ms (purple), compared to the traditional “diagonal” decoding method where a different classifier is trained and tested at the same time point (black). The thick lines indicate significant decoding scores. The yellow areas indicate when the diagonal performance was significantly different from the generalization across time. Error bars indicate the standard error of the mean (SEM) across subjects. (right) Generalization matrices. Decoding performance is plotted as a function of training time (vertical axis) and testing time (horizontal axis) for all classifiers. Decoding of the local-violation effect leads to a diagonal-shaped decoding performance from 82 ms to 508 ms (AUC over 50% in red), demonstrating that each classifier was only able to predict trials' classes for a short amount of time. Decoding of the global-violation effect leads to a square generalization matrix, suggesting that the underlying brain activity is essentially stable during this time period. Early classifiers of the global violation (<350 ms) are slightly lower than the traditional “diagonal” decoding performance, thus suggesting only a small change in the underlying pattern of activity.

The average event related fields (ERF) elicited by local auditory violations (local standard – local deviant) led, on average, to the traditional mismatch field, peaking at around 120 ms after the onset of the fifth sound (Figure 3, top left). The average ERF elicited by global auditory violations (global standard – global deviant) led to a sustained activity from ∼300 ms after the onset of the fifth sound (Figure 3, bottom left). Traditional analyses, including source reconstruction, are further detailed in [11], [15]. Note that traditional ERF analyses were applied across subjects, and are thus insensitive to inter-individual variability. In Figure 3, the topography of a single representative subject is plotted in comparison to the group average in order to highlight the potential loss of information induced by first order statistics applied at the group level.

A traditional “diagonal” decoding method, consisting in repeatedly training and testing a classifier with the MEG sensor data recorded at each time point, revealed the presence of decodable information between approximately 100 ms and 450 ms following the onset of the fifth sound (all p_FDR_<.05). Local auditory violations led to a decoding peak at 120 ms (AUC = 69.6%±7.9, p = .003) whereas global violations lead to a stable decoding performance from ∼150 ms to 700 ms (e.g. t = 350 ms: AUC = 66.3%±4.0, p = .003). This result confirms previous analyses showing a mismatch response around 120 ms [3], [15] and significant local and global effects ranging from 200 ms to 700 ms [7], [10], [11], [15].

Crucially, generalization-across-time demonstrated remarkably different dynamics for the local and global effects (Figures 3, top *versus* bottom). In the local contrast (decoding of local standards *versus* local deviants, corresponding to the classical mismatch response), none of the classifiers generalized over the full time window. Although the “diagonal” classifiers decoded information about local auditory novelty within a long time interval of approximately 400 ms, each classifier significantly generalized for ∼100 ms on average (p_FDR_<.05) and did not significantly differ from the corresponding diagonal classifiers over a time window of only ∼50 ms (p_FDR_<.05). Six classifiers, trained between 100 ms and 600 ms are presented in Figure 3 (top middle), and correspond to six lines of the temporal generalization matrix (Figure 3, top right). The results showed a clear diagonal pattern of temporal generalization and thus indicated that each classifier only generalized for a limited amount of time: each time sample was thus associated with a slightly different pattern of MEG activity. This result suggests that different brain regions are serially recruited, each for a short-lived time period, in response to a local auditory violation.

Interestingly, the classifiers trained around 120 ms generalized in the opposite direction around 200 ms. For example, a classifier trained at 114 ms led to a high AUC at this time point (AUC = 70.7%±7.2, p = .003), but generalized to an AUC below 50% at 200 ms (AUC = 34.3%±11.3, p = .003). This result means that trials were predicted to belong to the opposite class (*i.e.* standard trials were systematically predicted as deviant and *vice versa*). This below-chance performance suggests that the pattern of brain activity is inverted between these two time points. To test whether this reversal reflects the polarity reversal of a single pattern, the initial peak of diagonal decoding performance was compared to the peak of anti-generalization performance (*i.e.* AUC(t,t) versus (1-AUC(t,t′)) and *vice versa*. The results showed that diagonal performance was significantly higher than anti-generalization performance (F(8,1) = 5.75, p = .024). This result thus suggests that this reversal was only partial, and that a qualitatively different pattern of brain activity was elicited at 110 and 200 ms respectively. This hypothesis is further supported by the fact the diagonal decoding performance was always significantly above chance between these two time samples (all p<.004) whereas a simple polarity inversion would have led the diagonal decoding performance to drop to chance in the middle part of the reversal. However, as this pattern remains more complex than what was initially predicted, we would argue that only the late part of the diagonal (150–450 ms) unambiguously followed the simulation of serial processing (Figure 1, left).

Applying these analyses to the global contrast (global standard – global deviant) led to a strikingly different pattern of decoding performance. Within a broad temporal window, a nearly “square” pattern of temporal generalization indicated that most classifiers, regardless of their training time, produced very similar decoding performance across all testing times (Figure 3, bottom right). Decoding performance was statistically significant from approximately 125 ms until the end of the trial (700 ms). Sample classifiers trained between 100 ms and 600 ms and the full temporal generalization matrix are presented in Figure 3 (bottom middle). Overall, these findings show that a similar combination of MEG sensors can discriminate frequent auditory sequences from rare auditory sequences across many different time points. These results thus suggest that the underlying patterns of brain activity were sustained in a stable form for several hundreds of milliseconds. A weak but significant difference between the temporal generalization of the early classifiers (<350 ms, all p_FDR_<.05) and the traditional “diagonal” classifiers was also observed. This suggests that the early brain response to a global violation was partly changing over time, and became fully stable from 350 ms on.

## Discussion

We characterized the dynamics of the brain response to two types of auditory novelty detection. We predicted that *i)* local novelties should elicit a serial propagation of prediction error in successive brain areas whereas *ii)* global novelties should lead to an active maintenance of a particular pattern of brain activity. Traditionally, multivariate pattern classifiers are trained and tested at the same time point (*e.g.* [11], [46]–[49]) – an approach hereafter referred to as “diagonal decoding”. Here, by contrast, each classifier was trained to distinguish standard from deviant trials at distinct time sample, and evaluated their respective ability to generalize to all other time samples. The results showed that the two types of auditory violations are characterized by strikingly distinct dynamics.

### Violation of a local auditory expectation leads to the serial progression of short-lived neural activity patterns

Decoding local-standard versus local-deviant trials revealed a diagonally-shaped pattern of temporal generalization, together with a partial reversal of decoding performance in an early time window.

The diagonally-shaped decoding performance shows that the topographical pattern of magnetic fields changes continuously over time. This novel result therefore suggests that the violation of a low-level auditory regularity successively and temporarily recruits a series of different brain areas and thus supports the early proposal that the low-level auditory novelties recruit several different generators [50].

Furthermore, this finding clarifies the neural mechanisms responsible for the detection of low-level auditory novelties. Indeed, studies based on a similar task and in combination with fMRI [4], [7], [51], intracranial EEG [7], [11] and source reconstruction of MEG recordings [15], had already shown that local auditory novelties elicit a strong BOLD and electric response in the vicinity of Heschl's gyrus and the underlying segment of the superior temporal gyri – including when the novelty consists in omitting the last sound [15]. While these studies characterized the anatomical location of the neuronal generators of the MMN, they did not investigate the extent to which distinct generators were serially activated, or, conversely, whether the MMN reflected the homogeneous activation of a single neural system.

More generally, the serial activations observed presently also fit with predictive-coding theories, which postulate that distinct brain regions compare internally generated predictions to the incoming bottom-up evidence [21]–[23]. Subtracting the sensory evidence and the prediction leads to a “prediction error” signal, which is passed on to higher areas that iteratively search for an internal model making sense of the incoming data. Empirical and modeling studies have shown that the MMN could reflect a prediction error [15], [20]. The results supplements this proposal by confirming that unexpected sounds lead to a serial propagation of brain activity.

Finally, the early reversal of decoding performance (“worse-than-chance” generalization between 120 ms and 200 ms) implies that the pattern of brain activity that distinguishes between standard and deviant sounds partly reverses between these time points. One interpretation is that the brain area(s) which is/are initially activated is/are subsequently inhibited (or *vice versa*). This interpretation fits with intracranial recordings [6], [7] and source reconstruction analyses [3], [15] which typically show a similar reversal in the primary auditory cortex. However, the physiological interpretation of this pattern remains ambiguous. For example, the above excitation/inhibition hypothesis is indistinguishable from an alternative hypothesis related to the reversal of currents flow. Indeed, if the neural currents first flow out the cortex (bottom-up) and then flow back in (top-down), the magnetic field would also reverse. Such a reversal may occur if an early feedforward prediction error signal is followed, in the same region, by a later top-down cancellation signal.

### Violation of a global auditory regularity leads to a single sustained activity pattern

In sharp contrast with the local-violation results, decoding global standard versus global deviant trials led to a nearly square-shaped temporal generalization matrix. This pattern results from the fact that whenever a classifier was trained at a given time sample, it generalized almost perfectly to any other informative time sample. This result thus suggests that the underlying neuronal activity is essentially stable from 200 to 700 ms approximately. In other words, a single sustained network of brain areas appears to be recruited and sustained during this time window. However, and although temporal stability is the dominant feature of the temporal generalization of the global contrast, a small but significant advantage along the diagonal compared with off-diagonal decoding performance (*i.e.* generalizing over time) was also observed between 200 and 350 ms. This pattern suggests that, during this period, a small temporal evolution of brain activity coexisted with the main effect of stable maintenance.

Interestingly, the global effect rose slightly later than the local violation one, and thus fits with the idea that this more abstract violation recruits higher levels of processing than the local novelties. Together with fMRI [7] and source analyses [15], these results also support the idea that this type of violation durably engages working memory resources allocated by the prefrontal, parieto-temporal cortex [52]–[54]. The meta-stable activity of this network has also been proposed as a hallmark of information broadcasting and conscious access [55]. It is unclear, however, whether the present activity corresponds to the content of working memory or to a more transient updating process.

### A systematic method to characterize the temporal dynamics of brain activity

Decoding in general and the present temporal generalization method in particular, present several advantages. With advances in neuroimaging, the number of brain signals that are recorded simultaneously increases rapidly and it becomes difficult to embrace all of the data at once. The present recordings were, for instance, obtained from 204 gradiometers and 102 magnetometers, each capturing different directions of the magnetic fields and their spatial gradients. Yet, the relationship between MEG sensors and brain areas dramatically varies as a function of subjects' anatomy and position in the scanner. Source analysis provides a way to put these different signals in a common space across subjects but suffers from strong methodological difficulties and often generates an even larger dimensionality problem than scalp analyses. Given these issues, and as discussed elsewhere (*e.g.* [11], [45]), the method of multivariate decoding followed by temporal generalization presents several major advantages. First, it combines all simultaneous recordings into a unique information estimate. Second, each classifier is fitted on a single subject separately, which maximizes sensitivity.

These two advantages are generic to decoding analyses. For example, in a previous study [11], we showed how multivariate pattern analyses could be applied to EEG, MEG and intracranial recordings to maximally detect the MMN and the P300b following an unexpected auditory stimulus. The results demonstrated that such techniques could be efficiently applied to individual subjects and thus allowed investigating clinical populations who often present abnormal EEG topographies and latencies because of brain and skull damages. In this case however, decoding techniques are used to detect brain activations *independently* of their underlying spatio-temporal properties. By contrast, we have shown here how decoding can be used to *characterize* the underlying neural dynamics evoked by unexpected sounds. Interestingly, and unlike source reconstruction, the dynamics of cortical activity can be identified without relying on the strong hypotheses associated with source reconstruction. Generalization across time analyses therefore provides a powerful supplement to traditional MEG analyses and paves the way to a systematic characterization of the dynamics subtending cognitive processes.
